# The Role of Butirprost^®^ as an Adjuvant in Enhancing the Effect of Antibiotics in Patients Affected by Chronic Bacterial Prostatitis: A Randomized Prospective Trial

**DOI:** 10.3390/medicina61010148

**Published:** 2025-01-17

**Authors:** Felice Crocetto, Armando Calogero, Michele Santangelo, Agostino Fernicola, Filippo Varlese, Benito Fabio Mirto, Fabio Machiella, Alfonso Falcone, Giovanni Pagano, Fabrizio Dinacci, Gaetano Giampaglia, Domenico Varriale, Francesco Trama, Salvatore Iaconis, Francesco Del Giudice, Gian Maria Busetto, Matteo Ferro, Francesco Lasorsa, Giuseppe Lucarelli, Ciro Imbimbo, Biagio Barone

**Affiliations:** 1 Department of Neurosciences, Sciences of Reproduction and Odontostomatology, University of Naples Federico II, 80131 Naples, Italy; felice.crocetto@unina.it (F.C.); fmirto22@gmail.com (B.F.M.); f.machiella@gmail.com (F.M.); alfonso.falcone01@gmail.com (A.F.); giovanni.pagano1@outlook.it (G.P.); fabriziodinacci18@gmail.com (F.D.); gaetanogiampaglia@hotmail.it (G.G.); domenicov93@libero.it (D.V.); salvatore.iaconis@gmail.com (S.I.); ciro.imbimbo@unina.it (C.I.); 2 Section of General Surgery, Department of Advanced Biomedical Sciences, University of Naples Federico II, 80131 Naples, Italy; armando.calogero2@unina.it (A.C.); michele.santangelo@unina.it (M.S.); agostino.fernicola@unina.it (A.F.); filippo.varlese@unina.it (F.V.); 3 Urology Complex Unit, ASL Napoli 2 Nord ‘Santa Maria delle Grazie’ Hospital, 80078 Pozzuoli, Italy; francescotrama@gmail.com; 4 Department of Urology, Policlinico Umberto I, Sapienza University of Rome, 00185 Rome, Italy; francesco.delgiudice@uniroma1.it; 5 Department of Urology and Organ Transplantation, University of Foggia, 71122 Foggia, Italy; gianmaria.busetto@unifg.it (G.M.B.); francesco-lasorsa96@libero.it (F.L.); 6 Division of Urology, European Institute of Oncology (IEO) IRCCS, 20139 Milan, Italy; matteo.ferro@ieo.it; 7 Urology, Andrology and Kidney Transplantation Unit, Department of Precision and Regenerative Medicine and Ionian Area-Urology, University of Bari “Aldo Moro”, 70121 Bari, Italy; giuseppe.lucarelli@inwind.it; 8 Department of Urology, 196152 Ospedale San Paolo, ASL NA1 Centro Naples, 80145 Naples, Italy

**Keywords:** bacterial prostatitis, antibiotics, LUTS, QoL, IPSS, hyaluronic acid

## Abstract

Bacterial prostatitis (BP) is a common prostatic infection characterized by pain and urinary symptoms, often with negative bacterial cultures from prostatic secretions. It affects young and older men bimodally and impacts quality of life (QoL) significantly. *Background and Objectives*: Treatment typically involves antibiotics, but a multimodal approach with additional nutraceuticals may enhance outcomes. This study aimed to assess the efficacy of Butirprost^®^ in association with fluoroquinolones in patients with chronic bacterial prostatitis (CBP). *Materials and Methods*: Patients diagnosed with prostatitis (positive Meares–Stamey test and symptom duration > 3 months) at the University of Naples “Federico II”, Italy, from March 2024 to July 2024 were included in this study. All patients underwent bacterial cultures. Patients were randomized into two groups: Group A received antibiotics plus Butirprost^®^ (sodium hyaluronate plus Plantago major) for one month, while Group B received antibiotics alone. International Prostatic Symptoms Score (IPSS) and National Institutes of Health Chronic Prostatitis Symptom Index (NIH-CPSI) questionnaires were administered at baseline and at 15 and 30 days. *Results*: Out of 60 patients (Group A: 30, Group B: 30), Group A showed significant improvement in IPSS and NIH-CPSI scores at 15 and 30 days compared to Group B. Notable improvements were observed in pain, urinary symptoms, and QoL. *Conclusions*: The administration of Butirprost^®^ along with fluoroquinolones resulted in a significant improvement in pain, urinary symptoms, and quality of life along with improvements in both IPSS and NIH-CPSI scores, in patients affected by chronic bacterial prostatitis compared with fluoroquinolones alone.

## 1. Introduction

Bacterial prostatitis (BP) represents one of the most common urological diseases among young and older men worldwide, being, usually, the first reason a urologist is consulted. Despite its prevalence, BP remains a significant and challenging health issue due to its complex etiology, varied clinical manifestations, and potential for recurrence [1,2]. Prostatitis affects a considerable portion of the male population, with estimates suggesting that it accounts for 8% to 10% of all outpatient urological visits. BP specifically accounts for 5–10% of all prostatitis cases, underscoring its importance as a recurrent and multifaceted health concern [3]. According to the National Institute of Diabetes and Digestive and Kidney Diseases (NIDDK), which operates under the National Institutes of Health (NIH), prostatitis is categorized into distinct classifications based on the nature and cause of the condition. These categories include acute bacterial prostatitis (Category I), chronic bacterial prostatitis (Category II), chronic prostatitis with inflammation or chronic pelvic pain syndrome (CP/CPPS, Category IIIa), chronic prostatitis without inflammation (Category IIIb), and asymptomatic inflammatory prostatitis (Category IV) [4,5]. Each of these classifications represents a different manifestation of the disease, with bacterial prostatitis falling into the acute and chronic categories. While acute prostatitis (ABP) is typically caused by the ascent of bacteria from the urethra, bladder, or rectum into the prostate and represents a complication of a urinary tract infection (UTI), where patients present with rapid-onset symptoms (fever, chills, dysuria, and pelvic pain), chronic bacterial prostatitis (CBP) is much more insidious, and it is often characterized by recurrent episodes of infections and ongoing discomfort in the pelvic region, without systemic symptoms like fever. Instead, patients may experience milder but persistent symptoms such as pelvic pain, urinary frequency, and discomfort during or after urination [6,7]. The most common pathogens associated with BP include *Escherichia coli*, which accounts for approximately 70–80% of ABP cases, and other uropathogens such as *Enterococcus faecalis*, *Klebsiella*, and *Proteus* species. In CBP, pathogens can be harder to identify, and recurrent infections are often caused by antibiotic-resistant strains of *E. coli* or other gram-negative bacteria, which makes the condition more challenging to treat effectively. As result, long-term antibiotic therapy is often required to prevent recurrence [1,8,9,10]. The use of proper antibiotic treatment is the gold standard in the management of acute and chronic bacterial infections: the first is treated according to the recommendations for complicated urinary tract infections, i.e., with cephalosporins or fluoroquinolones, while for the second, the use of fluoroquinolones is a first-line treatment [11]. The growing concern over antibiotic resistance has significantly impacted the treatment of bacterial prostatitis, especially chronic forms. The overuse of antibiotics globally has led to the rise of resistant strains of bacteria, making it increasingly difficult to treat recurrent infections effectively [12,13,14]. As a result, there is a strong push within the medical community to find alternative or adjunct therapies that can enhance the efficacy of antibiotics while reducing the overall reliance on these drugs [15,16,17]. In this context, several adjuvant agents have been proposed to improve the management of BP. These include anti-inflammatory medications to reduce pain and swelling, alpha-blockers to alleviate urinary symptoms by relaxing the muscles in the bladder neck and prostate, physical therapy to address pelvic floor dysfunction, and nutraceutical agents aimed at enhancing the body’s natural defenses and promoting healing [18]. Nutraceuticals, in particular, have garnered significant attention for their potential role in managing inflammatory conditions, including bacterial prostatitis. These agents, derived from natural sources, are believed to offer a range of therapeutic benefits with minimal side effects. Their appeal lies not only in their anti-inflammatory and antioxidant properties but also in their potential to complement conventional treatments, thereby reducing the duration and dosage of antibiotic therapy required. This is especially important in an era where antibiotic stewardship is critical in preventing the further spread of resistant bacterial strains [19]. One such nutraceutical that has shown promise in the management of bacterial prostatitis is sodium hyaluronate. This compound, which is a derivative of hyaluronic acid (HA), has been extensively studied for its role in managing various inflammatory diseases [20,21,22]. HA is a naturally occurring glycosaminoglycan found in the extracellular matrix of connective tissues, where it plays a crucial role in maintaining tissue hydration, elasticity, and integrity [23]. Its ability to modulate inflammatory responses and promote tissue repair makes it an attractive option for treating conditions characterized by chronic inflammation, such as CBP [24]. Hyaluronic acid is composed of repeating disaccharide units of N-acetylglucosamine (GlcNAc) and glucuronic acid (GlcUA), linked together via alternating β-1,4- and β-1,3-glycosidic bonds. In the body, HA serves as a major component of synovial fluid and cartilage, where it protects articular cartilage from damage and plays a central role in joint lubrication. These properties have led to its widespread use in the treatment of osteoarthritis and other joint disorders [25,26]. However, emerging evidence suggests that HA, particularly in the form of sodium hyaluronate, may also have applications in the treatment of urological diseases, including bacterial prostatitis [27,28]. The use of sodium hyaluronate in urology is primarily based on its ability to enhance the body’s natural healing processes while reducing inflammation. Studies have demonstrated that this compound can improve the efficacy of antibiotics when used in combination therapy, particularly in the treatment of chronic bacterial prostatitis [29,30,31]. It is believed that by reducing inflammation and promoting tissue repair, sodium hyaluronate can help alleviate the symptoms of prostatitis and reduce the likelihood of recurrent infections. Moreover, its use is associated with minimal adverse effects, making it a safe and well-tolerated option for patients who may be sensitive to other medications [32].

Another promising nutraceutical for the management of BP is Plantago major, commonly known as broadleaf plantain. This medicinal plant has been used in traditional medicine for its anti-inflammatory, antimicrobial, and antioxidant properties, which make it a valuable adjunct in treating chronic inflammatory conditions such as prostatitis [33]. The bioactive compounds found in Plantago major, such as aucubin, flavonoids, and polysaccharides, have demonstrated potent anti-inflammatory effects, which could help reduce the inflammation associated with prostatitis. This reduction in inflammation may alleviate pelvic pain and improve urinary symptoms commonly experienced by patients with CBP and chronic pelvic pain syndrome (CPPS) [34]. Additionally, the antimicrobial properties of Plantago major are noteworthy, particularly in the context of bacterial prostatitis, where microbial infections play a central role. Some studies have indicated that aucubin, a compound in Plantago major, exhibits antibacterial effects against various bacterial strains. This property could enhance the efficacy of antibiotics when used in combination therapy, potentially reducing the recurrence of infections in patients with CBP. Moreover, the antioxidant effects of Plantago major, attributed to its rich content of flavonoids and phenolic compounds, may help counteract the oxidative stress that often accompanies chronic inflammation in the prostate [35,36].

In light of these findings, attention has recently shifted to a new nutraceutical formulation called Butirprost^®^, which includes sodium hyaluronate and Plantago major in the form of suppositories for the treatment of CBP. The present study aims to evaluate the efficacy of this combined therapy with fluoroquinolones and nutraceuticals in treating patients with CBP, offering a potential new approach to managing this challenging and recurrent condition.

## 2. Materials and Methods

We designed a prospective, non-blinded, randomized controlled trial (RCT) that aimed to evaluate the efficacy of combination therapy involving fluoroquinolones and Butirprost^®^ in patients with chronic bacterial prostatitis (CBP). This trial enrolled consecutive patients who attended the Urology Clinic at the University of Naples “Federico II” over a specific period from March 2024 to July 2024. The study adhered to the principles outlined in the Declaration of Helsinki and Good Clinical Practice (GCP) guidelines. All participants provided written informed consent before taking part in the study, and the study protocol was approved by the University of Naples “Federico II” ethical review board (document number 156/2023, approved on 28 February 2024). The study has been registered on ClinicalTrials.gov (accessed on 15 November 2024) with the following identification: NCT06684626.

The diagnosis of CBP was established based on the disease’s clinical definition, which required symptoms lasting for over three months, including dysuria, pelvic pain, and/or discomfort. Additionally, a positive Meares–Stamey test result was essential for confirming the diagnosis. The inclusion criteria were the following: patient aged between 18 and 50 years, symptoms consistent with CBP, and positive Mears–Stamey test. The exclusion criteria of the study were the following: patients younger than 18 years, history of neurological disease, urinary stones or cancer, allergy to fluoroquinolones or any components of Butirprost^®^ (ADL Farmaceutici, Milan, Italy), post-void residual > 50 mL, use of alpha-blockers or 5-alpha-reductase inhibitors (5-ARI), previous prostatic surgery, antibiotic treatment within four weeks prior to the study, refusal to provide informed consent, and incomplete follow-up data. Patients testing positive for certain pathogens like Chlamydia trachomatis (Ct), Ureaplasma urealyticum, Neisseria gonorrhoeae, herpes simplex virus types 1 and 2 (HSV-1/2), and human papillomavirus (HPV) were also excluded from the study. Only patients with uropathogens such as enteric gram-negative rods, enterococci, Staphylococcus saprophyticus, and group B streptococci were included in the trial. Excluded uropathogens represent a less common cause of CBP that requires different treatment approaches and may not respond similarly to the fluoroquinolone therapy used in this study.

Patients were randomized 1:1 via simple randomization utilizing desktop software to generate a random sequence of numbers. According to the group, the treatment schedule was based on fluoroquinolone (levofloxacin, 1 tablet 500 mg daily for 4 weeks) plus 1 tablet of Butirprost^®^ daily for 4 weeks (Group A) or oral fluoroquinolone alone (Group B). The Meares–Stamey test was repeated one month after the completion of therapy to assess bacterial eradication and evaluate the effectiveness of the treatment in each group. Additionally, patients were asked to complete two validated questionnaires at different points in the study: the National Institutes of Health Chronic Prostatitis Symptom Index (NIH-CPSI) and the International Prostate Symptom Score (IPSS), using the Italian versions of both. Patients completed these questionnaires at the beginning of the study (baseline), and follow-up assessments were scheduled 15 and 30 days after the start of therapy.

### 2.1. Composition and Characterization of Butirprost^®^

Butirprost^®^ is a nutraceutical formulation in suppository form, designed for the management of chronic bacterial prostatitis (CBP). It contains key ingredients such as Polyethylene Glycol (PEG) 1500, sodium hyaluronate, Plantago major, and ananas dry extract. PEG 1500 (94.1 g/100 g) serves as a base for the formulation. Sodium hyaluronate (0.1 g/100 g), a derivative of hyaluronic acid (HA), is valued for its potent anti-inflammatory and tissue-regenerative properties. Plantago major (5 g/100 g) contributes its soothing and wound-healing effects. Ananas dry extract (0.8 g/100 g) acts as an antioxidant in the formula, enhancing the overall therapeutic efficacy of the formulation in treating CBP.

### 2.2. Microbiological Diagnosis and Sensitivity Testing

The diagnosis of chronic bacterial prostatitis was supported by the Meares–Stamey test, a well-established diagnostic procedure. This test was performed as follows:

- Initial Urine Sample (Sample 1): A clean-catch midstream urine sample was collected to assess the presence of any initial urinary tract infection (UTI).
- Prostatic Massage: A digital rectal exam (DRE) was performed by the urologist to stimulate the prostate and release prostatic fluid.
- Post-Massage Urine Sample (Sample 2): A second urine sample was collected immediately after the prostate massage to detect bacterial presence or increased white blood cells.
- Expressed Prostatic Fluid (Sample 3): Prostatic fluid expressed during the DRE was collected and analyzed for bacterial growth and inflammatory markers.
- Post-Void Urine Sample (Sample 4): A final urine sample was obtained post-void to evaluate for residual bacteria or inflammatory markers.

These samples were processed using standard microbiological procedures. Samples were inoculated onto appropriate culture media, incubated under optimal conditions, and analyzed to identify bacterial pathogens. The identification of microorganisms was performed using standard techniques, including Gram staining, biochemical tests, and, where applicable, MALDI-TOF mass spectrometry. Antibiotic sensitivity testing was conducted using the disk diffusion method (Kirby–Bauer) or automated systems (e.g., VITEK 2, bioMérieux Italia Spa, Bagno a Ripoli, Italy) to determine the susceptibility of isolated pathogens to fluoroquinolones and other relevant antibiotics. While these microbiological and sensitivity testing methods were integral to confirming the bacterial etiology of chronic bacterial prostatitis and guiding therapy, the focus of this study was on evaluating the clinical outcomes of the combination therapy. For this reason, detailed descriptions of these methodologies were considered beyond the scope of the manuscript. These procedures were performed by qualified microbiologists adhering to standard diagnostic protocols.

### 2.3. Statistical Analysis

Descriptive statistics were presented as means and standard deviations for continuous variables, while categorical variables were expressed as frequencies and percentages. The Kolmogorov–Smirnov test was used to assess the normality of the data. The sample size calculation was based on the IPSS, with an alpha level of 0.05, a power of 80%, and anticipated mean scores of 8 ± 3.5 for Group A and 6 ± 1.5 for Group B, resulting in a required sample size of 60 participants. Group comparisons were conducted using the independent-sample Mann–Whitney U test for continuous variables and the Chi-square test for categorical variables. All statistical analyses were performed using IBM SPSS software (version 25, IBM Corp., Armonk, NY, USA), with a significance level set at *p* < 0.05.

## 3. Results

A total of 60 patients were enrolled in this study, with 30 participants assigned to Group A and 30 to Group B, based on the established eligibility criteria. The most frequently isolated bacteria included Escherichia coli (80%), Enterococcus faecalis (10%), Proteus mirabilis (8%), and Klebsiella pneumoniae (2%). Descriptive statistics for the cohort are summarized in Table 1. The two groups were comparable in terms of age (38.53 ± 4.5 years for Group A vs. 38.87 ± 4.65 years for Group B, *p* = 0.778). Initial IPSS scores (13.27 ± 1.964 for Group A vs. 13.43 ± 1.612 for Group B, *p* = 0.721) and NIH-CPSI total scores (29.6 ± 5.593 for Group A vs. 28.5 ± 6.642 for Group B, *p* = 0.491), as well as the respective subsets, did not differ significantly between the groups at baseline (Table 1). After one month, no patients tested positive according to the Meares–Stamey test. At 15 days, the IPSS score was 10.43 ± 2.991 for Group A compared to 12.00 ± 2.421 for Group B (*p* = 0.030). The NIH-CPSI total score was 21.6 ± 5.593 for Group A and 25.33 ± 6.216 for Group B (*p* = 0.018). Within the IPSS QoL domain, scores were 3.30 ± 1.685 for Group A versus 4.10 ± 1.242 for Group B (*p* = 0.041). For the NIH-CPSI subsets, the pain domain scored 13.20 ± 1.864 for Group A compared to 14.57 ± 2.515 for Group B (*p* = 0.200), while the urinary and QoL domains scored 4.20 ± 1.864 vs. 5.57 ± 2.515 (*p* = 0.020) and 4.20 ± 1.864 vs. 5.20 ± 1.864 (*p* = 0.042), respectively. After one month, the IPSS score was significantly lower in Group A (5.33 ± 2.057) compared to Group B (8.33 ± 2.057) (*p* = 0.0001).

**Figure 1:**
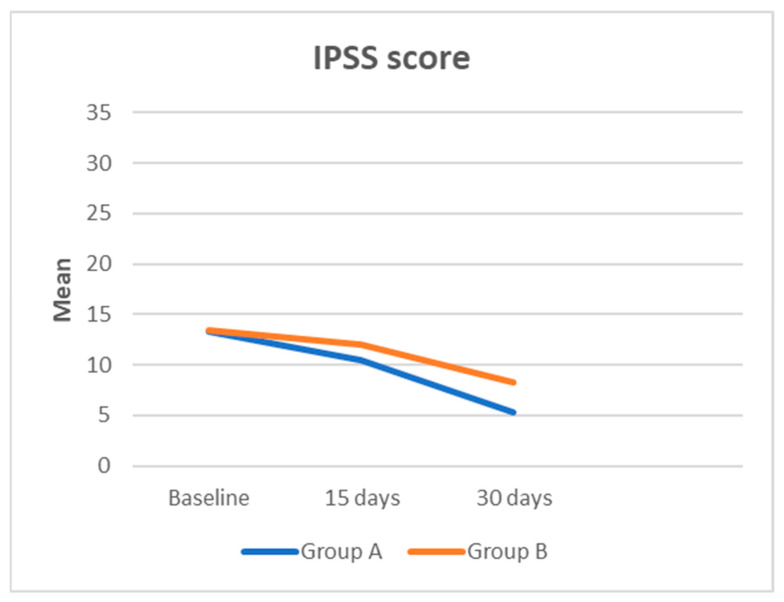
IPSS score from baseline to 30 days after treatment.

**Figure 2:**
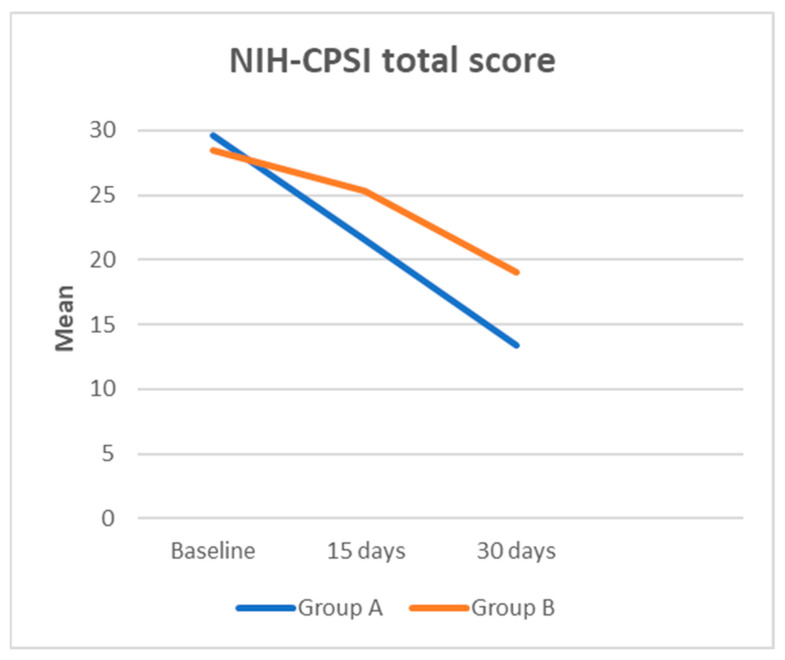
NIH-CPSI total score from baseline to 30 days after treatment.

The IPSS QoL score also showed significant improvement in Group A (1.00 ± 0.743) compared to Group B (2.23 ± 0.935) (*p* = 0.0001). The NIH-CPSI total score was 13.37 ± 2.895 for Group A versus 19.07 ± 3.331 for Group B (*p* = 0.0001). In the NIH-CPSI pain domain, Group A scored 11.20 ± 1.864 compared to 13.20 ± 1.864 in Group B (*p* = 0.0001). Similarly, the urinary and QoL domains of the NIH-CPSI after one month were 1.07 ± 0.740 vs. 2.93 ± 1.015 (*p* = 0.0001) and 1.10 ± 0.803 vs. 2.93 ± 1.015 (*p* = 0.0001), respectively (Figure 1 and Figure 2; Table 2). Among the 60 patients, no severe adverse effects were reported for either Butirprost^®^ or fluoroquinolones. Minor side effects related to fluoroquinolone therapy, such as mild gastrointestinal discomfort or transient headache, were noted in a small number of participants (n = 6), but these did not necessitate discontinuation of the treatment. No adverse effects specifically attributable to Butirprost^®^ were reported.

## 4. Discussion

CBP represents a persistent, common, and wide-spread challenge in urological practice, characterized by recurrent bacterial infections and pelvic discomfort. Although conventional therapies and antibiotic therapies are the mainstays of treating this condition, a proportion of patients do not respond to treatment despite reporting negative prostatic fluid cultures [37,38]. The complexity of CBP arises from various factors and encompasses a multifactorial etiology, difficulties in drug delivery to the prostatic tissue, and the recurrence of the condition. Indeed, it has been proposed that chronic prostatitis may represent an inflammatory dysregulation in response to persistent chemokine upregulation, oxidant stress, and cellular injury [39,40]. If an initial diagnosis of chronic prostatitis is established, it is evident that the condition is not a single disease entity, as different factors may be involved even in a single patient [41]. The use of antibacterial agents may not achieve the complete eradication of the infection due to multifactorial reasons, such as the poor distribution of drugs to the prostatic tissue, inadequate duration of antibiotic therapy, or chemical modification in situ, which could impair the efficacy of the delivered treatment. In particular, the formation of bacterial biofilm, i.e., a structure formed by microbial communities attached with substratum and embedded in a self-produced non-crystalline extracellular polymeric matrix, further impairs the efficacy of antibiotic therapy due to the protection provided by this structure to microbial communities against the antibiotic itself and the immune cells, thus providing a biological niche, which permits the recurrence of the infection [42,43,44]. The use of nutraceutical agents in the treatment of chronic prostatitis has gained increased popularity due to their unique mechanism of action, low side effect profiles, and high level of acceptance among patients. As reported in other studies, the possibility of using naturally extracted compounds in association with antibiotic therapy could improve the efficacy in terms of symptom relief and recurrence reduction [45,46,47]. The present study aimed to explore whether the combination of fluoroquinolones with Butirprost^®^, a nutraceutical formulation containing sodium hyaluronate and Plantago major, would improve clinical outcomes for patients with CBP compared to antibiotics alone. The findings suggest that this combination therapy yielded significantly better outcomes in terms of symptom relief, management of pain, and quality of life compared to antibiotics alone. Specifically, patients in Group A, who received the combination of fluoroquinolones and Butirprost^®^, exhibited substantial reductions in IPSS and NIH-CPSI scores compared to those in Group B, who were treated with antibiotics alone. These results highlight the potential of Butirprost^®^ to serve as a beneficial adjunct to conventional antibiotic therapy for the management of CBP. One of the primary mechanisms by which Butirprost^®^ appears to exert its effects is through the anti-inflammatory and tissue-regenerative properties of sodium hyaluronate [24,48]. Hyaluronic acid (HA) has been widely used in treating inflammatory conditions due to its role in tissue hydration, elasticity, and the modulation of inflammatory responses. The results of this study suggest that these properties extend to the urological domain, where HA, particularly in its sodium hyaluronate form, may help reduce the persistent inflammation seen in CBP. Additionally, Plantago major plays a crucial role in augmenting the therapeutic benefits. Plantago major is indeed known for its anti-inflammatory, wound-healing, and soothing properties, which are especially valuable in urological inflammatory conditions like CBP [49,50,51]. By reducing oxidative stress and promoting tissue repair, Plantago major contributes to the overall anti-inflammatory effects of Butirprost^®^. By enhancing tissue repair and mitigating inflammation, Butirprost^®^ may create a more favorable environment for antibiotic therapy to act, leading to better symptom relief and lower infection recurrence rate. Previous studies have highlighted the difficulty of treating CBP due to factors like bacterial biofilm formation, which can shield pathogens from antibiotics and immune responses [12,42,46,52,53,54]. Bacterial biofilms, particularly in the prostate, present a significant challenge as they reduce drug penetration and create a reservoir for recurrent infections [55,56]. The findings of our study align with the growing body of literature that suggests adjunct therapies targeting inflammation and biofilm disruption may be critical in managing chronic bacterial infections. Moreover, the inclusion of other bioactive compounds in Butirprost^®^, such as sodium hyaluronate and Plantago major, may also contribute to the observed improvements. The beneficial effects of the bioactive compounds included in Butirprost^®^ may explain why patients in Group A experienced significantly greater relief from urinary symptoms and pelvic pain compared to those receiving antibiotics alone. However, it is important to note that our findings were based on indirect data, and changes in inflammatory mediators and bacterial biofilms were not assessed directly in this study. While our primary focus was on clinical symptom improvement as measured by NIH-CPSI and IPSS scores, we recognize that understanding the underlying mechanisms, particularly the role of inflammatory mediators, could provide valuable insights into the therapeutic effects of Butirprost^®^. Future research should explore these aspects to further elucidate the anti-inflammatory and tissue-regenerative properties of this nutraceutical. The growing concern over antibiotic resistance further emphasizes the importance of exploring adjunct therapies in the management of chronic bacterial infections. The overuse of antibiotics has led to the emergence of resistant bacterial strains, making it increasingly difficult to treat recurrent infections effectively. Nutraceuticals like Butirprost^®^ offer a promising solution by potentially reducing the duration and dosage of antibiotic therapy required. In this context, the results of this study are particularly relevant, as they suggest that combining antibiotics with nutraceuticals may offer a more sustainable approach to managing CBP, one that addresses both the bacterial and inflammatory components of the disease.

Despite the promising findings, this study is not without limitations. The relatively small sample size of 60 participants, while sufficient to demonstrate statistical significance, limits the generalizability of the results. Additionally, the study was non-blinded, which may introduce bias, particularly in self-reported outcome measures like the NIH-CPSI and IPSS scores. One of the key limitations also lies in the debated decision to not include a placebo or no-treatment control group in our experimental design. While such a control group could have provided valuable insights into the baseline effects of treatment, ethical and practical considerations influenced our approach. Withholding active treatment in CBP, which impacts patients’ quality of life, raised ethical concerns about undue suffering. Additionally, including a placebo or no-treatment group could have led to higher dropout rates due to persistent symptoms, potentially compromising the study’s validity. Instead, comparing fluoroquinolone monotherapy with the combination therapy of fluoroquinolones and Butirprost^®^ allowed us to ensure that all participants received effective treatment while evaluating the incremental benefits of the nutraceutical. Future studies should aim to include larger, more diverse patient populations, ideally in a double-blind, placebo-controlled design, to confirm these findings. Longer follow-up periods would also be beneficial to assess the long-term efficacy of combination therapy with Butirprost^®^ and fluoroquinolones, particularly in preventing recurrent infections. Another limitation is the exclusion of patients with certain pathogens, such as Chlamydia trachomatis and Ureaplasma urealyticum, which represent a rare cause of prostatitis in some individuals. Future research should explore the efficacy of Butirprost^®^ in broader patient populations, including those with atypical pathogens, to determine if the benefits observed in this study extend to other forms of prostatitis.

## 5. Conclusions

The combination of fluoroquinolones with the nutraceutical product Butirprost^®^ presents a promising therapeutic approach for the management of CBP. This study demonstrated that patients receiving this combination therapy exhibited significant improvements in clinical outcomes compared to those treated with antibiotics alone. The anti-inflammatory effect and tissue regenerative properties of sodium hyaluronate together with Plantago major appear to play a pivotal role in mitigating the persistent inflammation characteristics of CBP, contributing to symptom relief. These findings underscore the potential of integrating nutraceutical agents as adjuncts in the conventional treatment of CBP, particularly in an era where antibiotic resistance poses a growing concern. Further research with larger sample sizes and longer follow-up periods will be necessary to confirm these results and to explore the long-term benefits and mechanisms of action of such combination therapies.

## Figures and Tables

**Table 1:** Baseline characteristics of patients involved in the study.

Variables	Group A	Group B	*p*-Value
Age	38.533 ± 4.5	38.867 ± 4.65	0.7780
Weight	72.13 ± 3.137	73.10 ± 6.283	0.1180
Height	178.00 ± 4.426	177.93 ± 3.9212	0.9510
BMI	22.540 ± 1.0142	24.121 ± 1.5623	0.0570
BPM	70.30 ± 2.929	70.30 ± 2.950	0.9500
SaO_2_%	98.80 ± 0.997	98.73 ± 1.081	0.8050
IPSS score	13.27 ± 1.964	13.43 ± 1.612	0.7210
IPSS QoL domain	4.87 ± 1.106	4.73 ± 1.285	0.6680
NIH-CPSI total score	29.60 ± 5.593	28.5 ± 6.642	0.4910
NIH-CPSI pain domain score	16.20 ± 1.864	15.83 ± 2.214	0.4910
NIH-CPSI urinary domain score	7.20 ± 1.864	6.83 ± 2.214	0.4910
NIH-CPSI QoL score	6.20 ± 1.864	5.83 ± 2.214	0.4910

**Table 2:** Comparison of the two groups at 15 and 30 days after therapy.

		15 Days	*p*-Value	30 Days	*p*-Value
IPSS score	Group A	10.43 ± 2.991	0.0300	5.33 ± 2.057	0.0001
	Group B	12.00 ± 2.991		8.33 ± 2.057	
IPSS QoL score	Group A	3.30 ± 1.685	0.00410	1.00 ± 0.743	0.0001
	Group B	4.1 ± 1.242		2.23 ± 0.935	
NIH-CPSI total score	Group A	21.60 ± 5.593	0.0180	13.37 ± 2.895	0.0001
	Group B	25.33 ± 6.216		19.07 ± 3.331	
NIH-CPSI pain domain score	Group A	13.20 ± 1.864	0.0200	11.20 ± 1.864	0.0001
	Group B	14.57 ± 2.515		13.20 ± 1.864	
NIH-CPSI urinary domain score	Group A	4.20 ± 1.864	0.0200	1.07 ± 0.740	0.0001
	Group B	5.57 ± 2.515		2.93 ± 1.015	
NIH-CPSI QoL domain score	Group A	4.20 ± 1.864	0.0420	1.10 ± 0.803	0.0001
	Group B	5.20 ± 1.864		2.93 ± 1.015	

## Data Availability

The original contributions presented in this study are included in the article. Further inquiries can be directed to the corresponding author.
